# A multi-view graph contrastive learning framework for deciphering spatially resolved transcriptomics data

**DOI:** 10.1093/bib/bbae255

**Published:** 2024-05-27

**Authors:** Lei Zhang, Shu Liang, Lin Wan

**Affiliations:** Department of Control Science and Engineering, Tongji University, No. 4800 Cao’an Road, 201804, Shanghai, China; Shanghai Research Institute for Intelligent Autonomous Systems, Tongji University, Lane 55, Chuanhe Road, 201210, Shanghai, China; Department of Control Science and Engineering, Tongji University, No. 4800 Cao’an Road, 201804, Shanghai, China; Shanghai Research Institute for Intelligent Autonomous Systems, Tongji University, Lane 55, Chuanhe Road, 201210, Shanghai, China; Academy of Mathematics and Systems Science, Chinese Academy of Sciences, No. 55 Zhongguancun East Road, 100190, Beijing, China; School of Mathematical Sciences, University of Chinese Academy of Sciences, 19A Yuquan Road, 100049, Beijing, China

**Keywords:** spatially resolved transcriptomics, spatial domain identification, multi-view graph contrastive learning, graph augmentation, graph convolutional network, nonlocal dependency

## Abstract

Spatially resolved transcriptomics data are being used in a revolutionary way to decipher the spatial pattern of gene expression and the spatial architecture of cell types. Much work has been done to exploit the genomic spatial architectures of cells. Such work is based on the common assumption that gene expression profiles of spatially adjacent spots are more similar than those of more distant spots. However, related work might not consider the nonlocal spatial co-expression dependency, which can better characterize the tissue architectures. Therefore, we propose MuCoST, a **Mu**lti-view graph **Co**ntrastive learning framework for deciphering complex **S**patially resolved **T**ranscriptomic architectures with dual scale structural dependency. To achieve this, we employ spot dependency augmentation by fusing gene expression correlation and spatial location proximity, thereby enabling MuCoST to model both nonlocal spatial co-expression dependency and spatially adjacent dependency. We benchmark MuCoST on four datasets, and we compare it with other state-of-the-art spatial domain identification methods. We demonstrate that MuCoST achieves the highest accuracy on spatial domain identification from various datasets. In particular, MuCoST accurately deciphers subtle biological textures and elaborates the variation of spatially functional patterns.

## Introduction

Advances in spatially resolved transcriptomics (SRT) technology have transformed our ability to investigate gene expression patterns and cellular microenvironments in a spatially resolved manner [1, 2]. Sophisticated SRT sequencing platforms, such as 10X Visium, Stereo-Seq, Slide-Seq and others, provide us with high-resolution gene expression profiles and spatial locations [3]. SRT data comprise structural information from different perspectives, including spatially adjacent relationships and correlation of gene expression between cells or spots. The use of such structural information is vital for deciphering SRT data [4].

Significant strides have been made in deciphering the genomic spatial architectures of cells that make up tissues and cellular microenvironments [5, 6]. In particular, some methods were developed to analyze the similarity of gene expression among spatially adjacent spots. For example, Giotto uses the hidden Markov random field (HMRF) model to identify spatial domains with coherent gene expression patterns between spatially adjacent spots [7]. BayesSpace also employs the HMRF model to promote the clustering of spatially adjacent spots [8]. However, HMRF-based methods are computationally intractable when contending with large-scale data. Alternatively, graph neural networks (GNNs) [9] model the interrelationships among spatially adjacent spots and effectively mitigate the impact of technical noise by smoothing the gene expressions over spatially adjacent spots. SpaGCN uses a graph convolutional network (GCN) to integrate gene expression, spatial location and histology imaging data to identify spatial domains [10]. STAGATE employs a graph attention autoencoder framework to decipher spatial domains, and it integrates the preclustering of gene expressions to recharacterize the spatial dependency between spatially adjacent spots [11].

Recently, the contrastive learning (CL) framework has improved the performance of self-supervised learning in computer vision techniques [12–14]. Furthermore, by combining the advantages of GNNs and CL, graph contrastive learning (GCL) shows potential in improving the performance of SRT analysis methods. GraphST employs deep graph infomax (DGI) [13] to contrast the information between individual spots and their spatially adjacent spots [15]. ConST also utilizes DGI to contrast the information of spots at the local, global and contextual levels [16]. ConST uses the structural information of three levels, but only from the spatial perspective. SpaceFlow also uses DGI to contrast the information between spots and global representation [17]. In addition, SpaceFlow uses a regularization term to penalize spots spatially distant, but exhibiting similar embedding. In sum, existing methods are consensus-based in that spatially adjacent spots will display more similarity of gene expression compared witht spots in more distant areas [18].

However, these methods may have limitations when analyzing complex tissues that exhibit nonlocal spatial gene co-expression patterns. For example, in biological textures featuring laminar and horn structures, comparable gene expression profiles can arise over substantial spatial distances [19–21]. That is, nonlocal spots can have similar gene expression profiles. Using spots with similar gene expression profiles globally will help the model to capture structural dependence of these spots from a holistic perspective [22, 23]. In this way, the gene co-expression dependency coupled with spatially adjacent dependency, which we denoted as the dual scale structural dependency, would constitute the complete and sufficient structural information of SRT data. The DGI-based GCL framework can only contrast spots using a summary representation of a single structural dependency, making this framework inflexible and unable to adapt to multiple dependencies [24]. However, using the structural information of multiple graphs to generate relatively consistent representations is a significant challenge for GCL [25–29].

To address this gap, we advance the modeling of complex tissue architectures by developing MuCoST, an accurate, fine-grained and efficient computational framework for deciphering complex SRT data from dual scale structural dependency. To overcome the limitation of DGI-based methods for nonlocal spatial co-expression spots integration, we provide a multi-view GCL framework combining shared multi-view GCN autoencoder and InfoNCE [30] contrastive loss. Our contrastive framework allows to learn relatively consistent representation from spatially adjacent dependency and co-expression dependency, and encourages the model to learn discriminative representation from random expression. Unlike the DGI loss, which maximizes the mutual information between a spot and its neighbors summary only using the spatially adjacent dependency, InfoNCE can adaptively compare the cosine distance of spots between spatially adjacent dependency and co-expression dependency using random expression. Intuitively, the InfoNCE loss ensures that the spot representation of spatially adjacent dependency and co-expression dependency is more similar than that of spatially adjacent dependency and random representation, thus making the model learn more complete structural information. We apply MuCoST to benchmark on four datasets, including (1) the 12-slice of human dorsolateral prefrontal cortex dataset (10X Visium), (2) the coronal mouse brain dataset (10X Visium), (3) the mouse olfactory bulb dataset (Stereo-seq) and (4) the human breast cancer dataset (10X Visium). The results show that MuCoST is superior to the competing methods in terms of spatial domain identification accuracy and clustering representation of compactness and separability. In particular, MuCoST accurately captures subtle biological textures from fine-grained spatial domains, such as pyramidal layer, hippocampus, striatum and hypothalamus structures in the mouse brain and laminar organizations in the mouse olfactory bulb. MuCoST also reveals the variation of the spatial functional domains. By incorporating differential gene expression analysis and gene enrichment analysis, the learned spatial domains possess interpretability and exhibit specific biological functions. All the results show that MuCoST has advantages in deciphering and analyzing the complex spatial architecture of tissues, which helps to make meaningful biological discoveries.

## Results

### Overview of MuCoST

**Figure 1:**
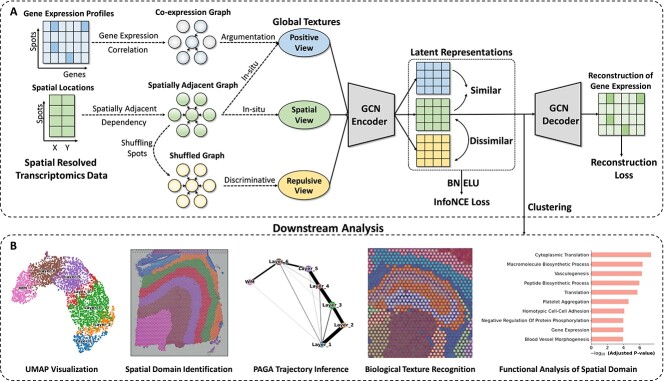
**Overview of MuCoST.** MuCoST is a multi-view GCL framework for deciphering SRT data. **A. Workflow of MuCoST.** MuCoST uses gene expression profile and spatial location information of SRT data as input data. Spatially adjacent graph, co-expression graph and shuffled graph are constructed in MuCoST. MuCoST adopts a multi-view GCL framework to learn latent representations of three views by the InfoNCE loss function. MuCoST extracts the compact latent representation through the reconstruction loss of GCN autoencoder. **B. Downstream analysis.** MuCoST performs clustering on latent representation of spatial view and realizes downstream analysis tasks, such as spatial cluster visualization, spatial domain identification, PAGA trajectory inference, subtle biological texture recognition and functional analysis of spatial domain.

MuCoST is a multi-view GCL framework for deciphering SRT data (Fig. 1A). MuCoST uses gene expression profile and spatial location information of SRT data as input. Co-expression graph, spatially adjacent graph and shuffled graph are constructed (Fig. 1A). The co-expression graph computes the gene expression correlations to capture the spatial-agnostic co-expression dependency, representing spots in the graph with globally similar expression regardless spatial information. Its purpose is to identify spots that are relatively far apart in space yet exhibit consistent gene expression. The spatially adjacent graph uses spatial locations to capture spatially adjacent dependency among spots that are spatially adjacent to each other in tissues, facilitating the gene representation similarity in spots with spatially adjacent locations. The shuffled graph is dynamically constructed in each training episode by randomly shuffling gene expression profile of spots from spatially adjacent graph.

MuCoST uses the above three graphs to construct contrastive views (Fig. 1A). The spatial view is composed of spatially adjacent graph for learning the *in situ* representation of spots. The positive view is an augmentation for spatially adjacent graph through co-expression graph to capture the global biological textures. The repulsive view is composed of shuffled graph which is used to adaptively learn discriminative embedding. MuCoST uses a shared GCN autoencoder to project the three views into latent representations (Fig. 1A). Using InfoNCE contrastive loss, MuCoST facilitates the similarity between the latent representation of spots in spatial view and positive view, allowing the model to effectively capture the structural information of spatially adjacent graph and co-expression graph. Furthermore, MuCoST induces a repulsive effect on the latent representation to promote dissimilarity between spatial view and the repulsive view (Fig. 1A). This repulsive effect guarantees that the spatial representation does not collapse and that it remains discriminative from random gene expression. In order to obtain the compact latent representation from the autoencoder, the reconstruction loss is utilized to reduce errors between the decoded representation and the input gene expression profile (Fig. 1A).

MuCoST detects spatial domains through clustering in the latent representation of the spatial view. Moreover, MuCoST can perform analytical tasks that include spatial cluster visualization, spatial domain identification, PAGA trajectory inference, subtle biological texture recognizing and functional analysis of spatial domain from spatial gene expression heterogeneity (Fig. 1B).

### MuCoST improves the performance of representation learning on human dorsolateral prefrontal cortex

**Figure 2:**
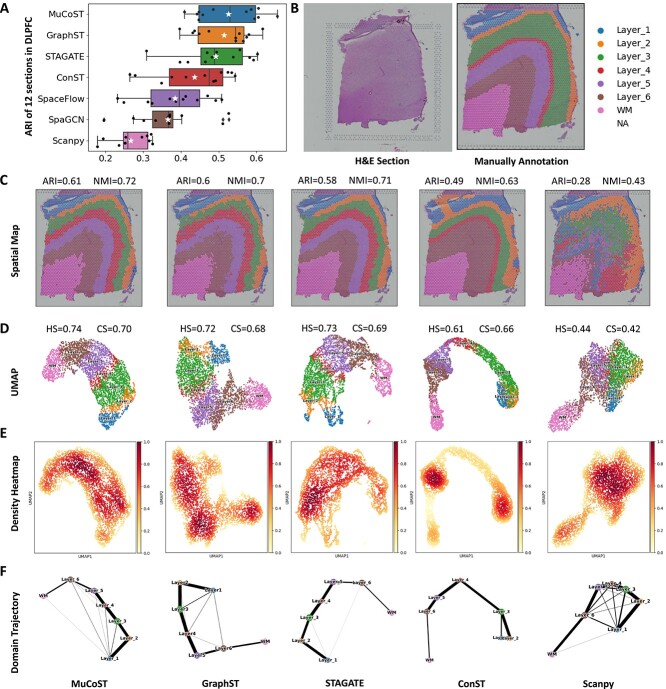
**MuCoST improves the performance of representation learning on human dorsolateral prefrontal cortex data.** **A.** Spatial domain identification results for MuCoST and competitive approaches in the human dorsolateral prefrontal cortex dataset with 12 sections. **B.** Slice #151673 of human dorsolateral prefrontal cortex data includes the H&E-stained section and manually annotated spatial domains. **C.** The results of spatial domain identification on slice #151673 demonstrated that MuCoST ranked among the highest scores compared with competitive methods. **D.** Clustering results were visually compared using UMAP. **E.** Compactness of clusters was visually compared through a density heatmap. **F.** The domain trajectory was compared visually using PAGA.

The precise deciphering of spatial architectures relies on the model’s learned representation. We first evaluated the representation learning performance of MuCoST on benchmark datasets. The 10X Visium dataset of the human dorsolateral prefrontal cortex (DLPFC) [20] is a widely used benchmark dataset for evaluating the performance of spatial clustering. This dataset comprises 12 slices, each slice with four or six DLPFC layers and one white matter (WM) layer. These domains have been annotated using morphological features and marker genes, resulting in a valuable resource for evaluating the clustering accuracy of the learned representations (Fig. 2B). We compared MuCoST with GraphST [15], STAGATE [11], ConST [16], SpaceFlow [17], SpaGCN [10] and Scanpy [31] for spatial domain accuracy across all 12 slices in DLPFC dataset (Fig. 2A, Figure S1) using adjusted Rand index (ARI), normalized mutual information (NMI), homogeneity score (HS), completeness score (CS) and V-measure. MuCoST achieved the best performance with the highest mean ARI accuracy of 0.526, GraphST achieved a mean ARI accuracy of 0.514 and STAGATE achieved a mean ARI accuracy of 0.492. None of the other methods surpassed the mean ARI accuracy of 0.45. (Fig. 2A). All detailed results of the 12 slices on DLPFC dataset are shown in Supplementary Figure S2.

To demonstrate the detailed performance of MuCoST, we provided spatial domain identification results for slice #151673, comprising six DLPFC layers and one WM layer (Fig. 2C–F). MuCoST achieved a maximum ARI score of $0.61$ and NMI of $0.72$. GraphST attained the second highest ARI score of $0.60$ and third highest NMI score of $0.7$, while STAGATE achieved the third highest ARI score of $0.58$ and the second highest NMI score of $0.71$ (Fig. 2C). ConST only identified five DLPFC layers ($\text{ARI}=0.49$ and $\text{NMI}=0.63$), whereas Scanpy failed to detect DLPFC layers 3 to 6 ($\text{ARI}=0.28$ and $\text{NMI}=0.43$). Additionally, to offer a comprehensive evaluation of MuCoST’s performance, we present a two-dimensional uniform manifold approximation and projection (UMAP) visualization of slice #151673 (Fig. 2D). MuCoST can distinguish between spots of different layers, achieving an HS score of $0.74$ and CS score of $0.7$ (Fig. 2D), while STAGATE achieved the second highest HS score of $0.73$ and CS score of $0.69$. Additionally, in the UMAP produced by STAGATE, the spots on layers 4 to 6 were mixed together, while the spots on layer 1 and layer 2 were separated into two subgroups (Fig. 2D). Similarly, GraphST did not properly separate the spots of layers 2 to 4. In terms of index score, GraphST got the third highest HS score of $0.72$ and CS score of $0.68$ (Fig. 2D). Furthermore, the spots of DLPFC layers 1 and 2 and 4 to 6 were mixed together in ConST, and Scanpy cannot distinguish among the spots of all domains (Fig. 2D). In addition, the density heatmap based on UMAP showed that MuCoST had a relatively uniform color depth, indicating that each cluster had consistent intra-class compactness (Fig. 2E, Figure S3). However, the color depth between clusters in ConST and Scanpy was quite different in that the spatial density between spots was not uniform (Fig. 2E). Moreover, the trajectory of spatial domains in ConST and Scanpy was inconsistent with layers in the spatial map. In contrast, MuCoST reflected the consistent correspondence between spatial domain trajectory and spatial map (Fig. 2F).

### MuCoST deciphers the subtle biological textures with fine-grained spatial domains of brain tissues

**Figure 3:**
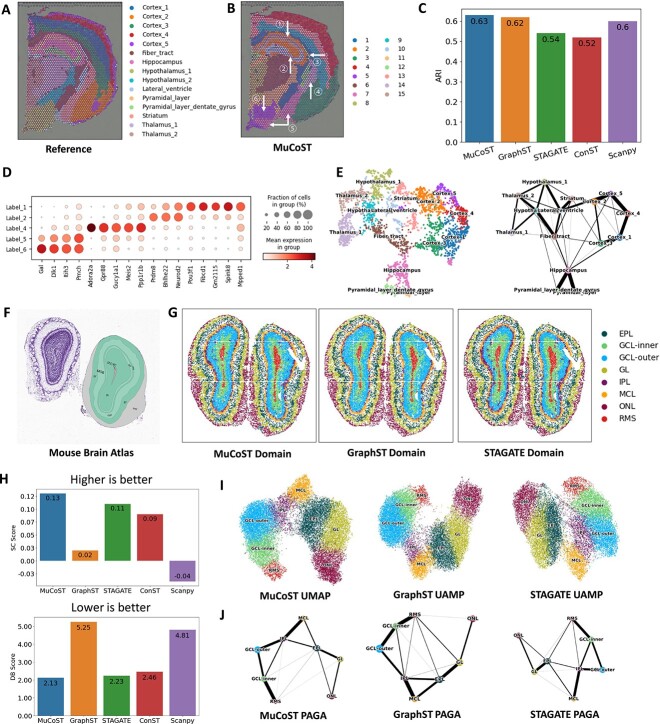
**MuCoST precisely identifies the subtle spatial architecture of brain tissues from SRT data.** **A.** Manually annotated spatial domains of mouse brain tissues. **B.** The spatial domains identified by MuCoST. **C.** ARI bars of five methods on spatial domain identification accuracy. **D.** Dotplot of spatial differential expression of genes. **E.** UMAP visualization and PAGA trajectory of clustering results. **F.** Reference atlas of mouse olfactory bulb. **G.** The spatial domains identified by three methods on mouse olfactory bulb. **H.** SC score (top) and DB score (bottom) bar of five methods on spatial cluster results. **I.** UMAP visualization of three methods on mouse olfactory bulb. **J.** PAGA trajectory of three methods on mouse olfactory bulb.

To illustrate the recognition ability of subtle biological textures using SRT data, we applied MuCoST to mouse brain tissues with subtle anatomical structures. Here, we use 10X Visium SRT data of coronal mouse brain slice, which are annotated by Allen Brain Atlas and Mouse Brain gene expression atlas in Squidpy [32] (Fig. 3A, Figure S6a). We found that the spatial domains identified by MuCoST using mclust clustering algorithm was highly consistent with the spatial domains manually annotated in Squidpy, and the highest identification accuracy was obtained (ARI=0.63, Fig. 3B and C). MuCoST accurately identified the subtle structures of Pytamidal_layer (Label_1) and Pyramid_layer_dentate_gyrus (Label_2) which are embedded in the Hippocampus, while GraphST confused these two structures as one layer, and STAGATE and Scanpy did not identify the embedded structure in Hippocampus. ConST identified the two subtle structures, but segmented the tail of the Pyramid_layer_dentate_gyrus (Fig. 3B, Figure S6b). MuCoST also retained a single-line Lateral_ventricle (Label_3) structure, which was not found in other competitive methods (Fig. 3B, Figure S6b). Compared with the annotation, MuCoST identified the spatial subdomain (Label_4) in Striatum, and from the dotplot of the differential expression of spatially variable genes, it can be found that there are spatially specific expression genes (Adora2a, Gpr88, Gucy1a1, Meis2 and Ppp1r1b) in Label_4 (Fig. 3B and D). These genes were found to be related to motor regulation, cognitive function and neurotransmitter regulation in the Striatum nucleus of Label_4 [33]. The nuclei in the spatial subdomain of Striatum (Label_5) identified by MuCoST was embedded in Hypothalamus_1, and the spatial subdomain of Hypothalamus_1 (Label_6) and Label_5 had clear differential expression patterns of spatially variable genes (Gal, Dlk1, Itih3 and Pmch, Fig. 3D). In addition, by comparing the mouse brain atlas, we found that the nuclei of spatial subdomain in Striatum (Label_5) had complex functional spatial domains, which was interconnected with Pallidum and embedded into hypothalamus (Figure S6a). We compared the spatial domain identification results of competition methods clustered by Leiden and Louvain algorithms (Figure S6c and d). MuCoST also obtained the highest identification accuracy, but missed subtle Pytamidal_layer structure. We found that Scanpy, which only used gene expression information without using spatial information, had higher identification accuracy than other competitive algorithms using spatial information, but its capture ability of subtle biological textures was not better than that using spatial information (Figure S6c and d). Finally, in order to eliminate the influence of different clustering algorithms, we analyzed the latent representation using ground truth spatial domains. Through UMAP visualization and PAGA trajectory analysis, it can be seen that MuCoST distinguished the spots in different spatial domain and aggregated the spots in each spatial domain, and found clear trajectory from PAGA (Fig. 3E). However, the spots of STAGATE and ConST were confused in UMAP visualization and did not show a clear trajectory (Figure S6e). In summary, MuCoST can effectively take advantage of co-expression dependence and spatially adjacent dependence information to achieve the best identification accuracy and subtle biological textures recognition.

Next, we applied MuCoST to sub-cellular spatial resolution Stereo-seq SRT data of mouse olfactory bulb tissue to study the identification of laminar structures (Fig. 3F). By performing a binning operation on spots, we achieved a cellular-level resolution of 14 um. From the Allen mouse brain atlas (https://mouse.brain-map.org/experiment/thumbnails/100048576?image_type=atlas) [21], the mouse olfactory bulb clearly shows laminar organizations, including olfactory nerve layer, glomerular layer (GL), external plexiform layer (EPL), mitral cell layer (MCL), internal plexiform layer (IPL), granule cell layer (GCL) and rostral migratory stream (RMS) (Fig. 3F). We applied five methods to learn the spatial domains of mouse olfactory bulb, and checked the consistency with the lamellar biological textures of reference atlas (Fig. 3G, Figure S7). MuCoST, GraphST and STAGATE identified consistent lamellar biological textures with fine-grained spatial domains (Fig. 3G). ConST identified the incomplete GCL layer, confused RMS layer and IPL layer, and confused GL and MCL layer. Scanpy cannot clearly identify lamellar biological textures (Figure S7). To quantitatively analyze the segmentation quality of lamellar biological textures, we calculated the Silhouette Coefficient (SC) score and Davies–Bouldin (DB) score of each method. The results show that MuCoST got the best score (SC=0.13, DB=2.13), GraphST, which also used CL framework, got a poor score (SC=0.02, DB=5.25) close to Scanpy (SC=-0.04, DB=4.81) without spatial information and STAGATE and ConST got similar scores, which were second to MuCoST (Fig. 3H). We further analyzed the recognition of lamellar biological texture from UMAP visualization and PAGA trajectory (Fig. 3I and J, Figure S7a and b). MuCoST, GraphST and ConST benefited from the repulsive view in the CL framework, and learned the distinguishing representation, presenting a U-shaped structure in UMAP visualization (Fig. 3I, Figure S7a). However, ConST had a tendency of feature collapse due to excessive use of spatial information, and the separation between adjacent clusters was not obvious (Figure S7a). STAGATE and Scanpy tended to cluster together (Fig. 3I, Figure S7b). In addition, MuCoST showed the development trajectory consistent with the lamellar biological textures, while the RMS layer of GraphST and STAGATE was obviously mixed with EPL and GL layers (Fig. 3J). This shows that MuCoST can achieve the best recognition performance of subtle biological textures in SRT data with spot resolution and cellular resolution through fine-grained spatial domain of brain tissues.

### MuCoST improves the interpretability of complex and heterogeneous cancer tissue with functional spatial domains

**Figure 4:**
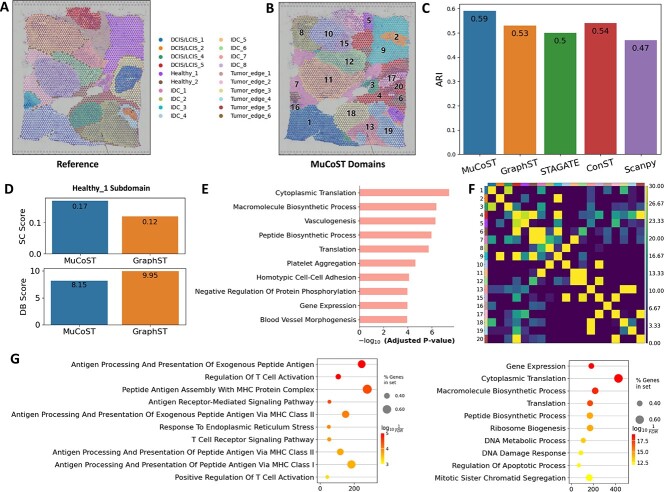
**MuCoST improves the interpretability of complex and heterogeneous cancer tissue with functional spatial domains**. **A**. A human breast cancer slice with manual annotation. **B**. Spatial domains annotation by MuCoST. **C**. Comparison of spatial domain identification accuracy with five methods. **D**. SC score and DB score bar chart of MuCoST and GraphST for Healthy_1 spatial subdomain. **E**. The enriched GO terms in spatial domain 2 relative to spatial domain 9. **F**. The interaction matrix between spatial domains. **G**. The enriched GO terms in spatial domain 1 and spatial domain 11.

To demonstrate the generalization ability in cancer tissue with complex spatial patterns and heterogeneous gene expressions, we tested MuCoST on the 10X Visium data of human breast cancer tissue. The human breast cancer dataset was annotated as 20 spatial domains, which were divided into four morphotypes: DCIS/LCIS, healthy, IDC and tumor edge [34] (Fig. 4A). In the task of spatial domain identification, MuCoST achieved the highest accuracy (ARI=0.59), which was nearly 10% higher than competitive methods (Fig. 4B and C, Figure S8a).

To illustrate the complexity of spatial pattern and high heterogeneity gene expression pattern of human breast cancer, we analyzed the spatial clustering in terms of spatial distance variation and spatially variable genes (Figure S8b–d). We computed the spatial pattern of clustered or dispersed spots distribution for each spatial domain based on Ripley’s L functions [32] (Figure S8b). We found three significant clustered spatial domains 11 (IDC_8), 1 (IDC_4) and 9 (Healthy_1) relative to other dispersed spatial domains (Figure S8b). We found that spatial domain 9 and spatial domain 2 were contained in Healthy_1, which was also found in GraphST, but the corresponding spatial domain 2 in GraphST was larger. STAGATE and Scanpy identified the spatial domain consistent with the reference, while ConST did not find a clear spatial pattern in Healthy_1 (Fig. 4B, Figure S8a). We calculated the SC score and DB score of two spatial domains in Healthy_1 of MuCoST and GraphST (Figure 4D). The results show that the clustering performance of MuCoST is better (high SC score, low DB score), and the gene expression visualization of marker genes were more consistent with the spatial domain 2 of MuCoST (Figure S8c). To further illustrate the function of spatial domain 2, we did gene ontology (GO) enrichment analysis [35] in spatial domain 2 versus spatial domain 9. In spatial domain 2, the pathways of cytoplasmic translation, vesculogenesis, platelet aggregation and blood vessel morphogenesis were significantly up-regulated, indicating that spatial domain 2 had angiogenesis-related functions. In the identification of spatial domain 1 and 11, MuCoST, GraphST and ConST were consistent with reference, while STAGATE and Scanpy identified multiple spatial subdomains. We analyzed the spatial patterns of spatial domain 1 and 11 through the interaction matrix (Fig. 4F). We found that 1 (IDC_4) interacted with surrounding spatial domains such as spatial domain 3 (IDC_1), 7 (Tumor_edge_2), 13 (IDC_2), 16 (IDC_3), 18 (DCIS/LCIS_4) and 19 (IDC_2) with various intensities, while 11 (IDC_8) only interacted significantly with spatial domain 7 (Tumor_edge_2) and 12 (IDC_6) (Fig. 4F). We screened the top 10 spatially variable genes with spatial domain 1 (IDC_4) versus 11 (IDC_8) (Figure S8d-left) and spatial domain 11 (IDC_8) versus 1 (IDC_4) (Figure S8d-right), respectively. We found that the genes significantly expressed in spatial domain 1 (IDC_4) participated in the process of tumor formation, invasion and metastasis (CCND1, S100A1, AGR2 and GFRA1), tumor inhibition (CXCL14) and cytoskeleton formation and regulation (KRT8 and TTLL12) (Figure S8d-left) [36]. However, the genes significantly expressed in spatial domain 11 (IDC_8) were involved in cell protection and repair (TFF3), immune response (IL6ST, SCGB2A2, SCGB1D1 and CFB) and maintaining cell function stability (H2AFJ, RAB11FIP1 and ERLIN2) (Figure S8d-right) [36]. From the main functions of these genes in spatial domains 1 (IDC_4) and 11 (IDC_8) and the enriched GO terms (Fig. 4G), we concluded that spatial domain 1 (IDC_4) staged the process of cancer cell invasion and tissue microenvironment inhibition, while spatial domain 11 (IDC_8) showed the trend of immune response and cell repair. This further explained that the spatial interaction of spatial domain 1 (IDC_4) in Fig. 4F was diverse, while the spatial interaction of 11 (IDC_8) was pure and simple. In summary, MuCoST promotes the understanding of cancer tissues with complex spatial patterns and gene expression heterogeneity through functional spatial domain analysis, which is also helpful for us to deepen our understanding of spatial architecture in SRT data.

### Ablation Study

We conducted a series of ablation experiments on DLPFC dataset to analyze and explain the function and mechanism of MuCoST.

- **MuCoST-w/o-ctr-spa** uses single spatially adjacent graph without CL framework.
- **MuCoST-w/o-ctr-coe** uses single co-expression graph without CL framework.
- **MuCoST-w/o-ctr-sce** uses the combination of spatially adjacent graph and co-expression graph without CL framework.
- **MuCoST-w/o-rep** uses CL framework with disable repulsive view (the spot representation is forced to zero).
- **MuCoST-w/-ctr-spa** uses CL framework with only spatially adjacent graph in positive view.
- **MuCoST-w/-ctr-coe** uses CL framework with only co-expression graph in positive view.

**Figure 5:**
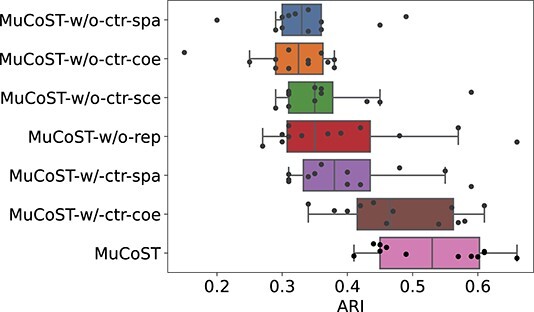
The ablation experiment results of MuCoST and its variants on DLPFC dataset.

The median ARI of MuCoST is better than its variant significantly, and the median ARI of the variants with CL framework outperform the variants without CL (Fig. 5).

Focused on #151673 of DLPFC (Figure S9), MuCoST-w/o-ctr-spa exhibited relative smoothness in the spatial domain, but the Layer_1 to Layer_4 were confused and identified as a single layer. MuCoST-w/o-ctr-coe could roughly distinguish the location of each layer, but the clustering was somewhat scattered. MuCoST-w/o-ctr-sce combined the advantages of both, identifying more layers than MuCoST-w/o-ctr-spa while reducing the scattered spots compared with MuCoST-w/o-ctr-coe. To emphasize the benefits of CL, we introduced a variant called MuCoST-w/o-rep, where the spot of the repulsive view is set to zero. This modification effectively disables the CL framework from extracting discriminative information from the repulsive view. Cluster 1 shows confusion between Layer_1 and Layer_5, while Layer_2 and Layer_4 are not successfully identified. It becomes evident that the absence of the repulsive view in MuCoST-w/o-rep hampers the CL framework’s ability to effectively capture distinct spatial patterns, leading to subpar performance in spatial domain identification. In the positive view using a single graph, MuCoST-w/-ctr-coe successfully identified six valid spatial domains, whereas MuCoST-w/-ctr-spa only identified five, mirroring the performance observed in the absence of the CL framework. However, by leveraging the combined strength of both spatially adjacent graph and co-expression graph in MuCoST, all seven spatial domains can be accurately identified.

## Discussion

The application of SRT technology allows us to attain high-resolution spatial maps of gene expression profiles, which can be utilized to uncover the spatial architectures of tissues. The complexity of spatial architectures is reliant on multiple internal data relationships in SRT data, including spatially adjacent relationships and correlation of gene expression. These perspectives provide complementary and redundant structural information in deciphering SRT data. Therefore, to integrate the above structural information, we proposed MuCoST, a multi-view graph CL framework, to learn representations from dual scale structural information. Using InfoNCE loss, MuCoST learned the consistent latent representation of spatially adjacent dependency and co-expression dependency. Furthermore, by shuffling gene expression profiles of spots in the repulsive view, MuCoST distinguished the *in situ* representation of spatial view from random expression. Thus, MuCoST can efficiently capture spatial architecture from gene expression and spatial information.

Drawing on the results of experiment, we concluded that MuCoST outperformed the competing methods in terms of clustering accuracy of solid benchmark and clustering representation of compactness and separability. Moreover, MuCoST exhibited exceptional performance in identifying ordered layers on DLPFC data, as exhibited by the capture of subtle spatial structures of pyramidal layer, hippocampus, striatum and hypothalamus on the coronal mouse brain dataset. MuCoST also showed superior performance and compatibility in Stereo-seq data with cellular resolution and demonstrated more precise laminar structures than competitive methods. MuCoST improved the interpretability of complex and heterogeneous cancer tissues through functional spatial domains. The ablation experiment showed that MuCoST ingeniously integrates the advantages of various components in its variants, ultimately achieving higher performance and stability in spatial pattern identification and other downstream analysis. In addition, the robust analysis of 12 slices from DLPFC datasets showed that MuCoST converged quickly and remained stable for different random seeds (Figure S4a and c). When setting the temperature of InfoNCE loss function, MuCoST could easily adapt to lower temperature on 12 slices from DLPFC datasets (Figure S4b). Finally, multi-view graph CL showed promise in combining various positive data, and in subsequent research, we will investigate the integration of multi-batches and multi-omics spatial genomics data.

## Methods

### Data description

We benchmark MuCoST with SRT data of brain and cancer tissues, which come from 10X Visium and Stereo-seq. More specifically, DLPFC tissues of 10X Visium dataset include 12 slices, each of which has five to seven layers of DLPFC and WM [20]. For mouse brain tissues of 10X Visium dataset, the fine-grained spatial domains are annotated in Squidpy by Allen mouse brain atlas and mouse brain gene expression atlas [32]. The mouse olfactory bulb tissue of Stereo-seq dataset is binned into a cellular resolution of 14 um. Human breast cancer tissue of 10X Visium dataset is annotated into 20 spatial domains by SEDR [34].

### Data preprocessing

We use the Scanpy package, specifically utilizing the Seurat v3 algorithm, to screen highly variable genes across 3000 dimensions of gene expression profiles. Following screening, we perform a standard normalization, log-transformation and scaling procedure for subsequent processing and analysis.

### Constructing graphs

MuCoST constructs three graphs using SRT data, and these graphs provide distinct perspectives on the underlying biological spatial architectures within tissues.

#### Spatially adjacent graph

The spatially adjacent graph $\mathbf{G}_{\text{spa}}(\mathbf{X}, \mathbf{E}_{\text{spa}})$ is the basic view of SRT data. It consists of gene expression profile $\mathbf{X}$ and the edge list of spatially adjacency graph $\mathbf{E}_{\text{spa}}$. $\mathbf{E}_{\text{spa}}$ is determined by the spatial coordinates of each spot, and each spot is connected to its maximum of $k$ nearest neighbor spots. Specifically, in 10X Visium data, the construction of $\mathbf{E}_{\text{spa}}$ is based on a radius parameter, which is used to determine the $\epsilon -$nearest neighbors for each spot in the SRT dataset (we set $\epsilon =150$ as default). We ensure that each spot is linked with a maximum of $k=6$ neighboring spots to accurately depict spatial relationship. However, for other sequencing platforms such as Stereo-seq, we use the $k-$nearest neighborhood algorithm to calculate spatial neighbors, owing to the uneven spatial density of spots measured by this platform.

#### Co-expression graph

The co-expression graph $\mathbf{G}_{\text{coe}}(\mathbf{X}, \mathbf{E}_{\text{coe}})$ is constructed using only gene expression profile. It consists of gene expression profile $\mathbf{X}$ and the edge list of co-expression graph $\mathbf{E}_{\text{coe}}$. $\mathbf{E}_{\text{coe}}$ is constructed using cosine similarity of gene expression profiles of each spot. Because of complex calculation, the gene expression profile is reduced to latent dimension using a principal component analysis (PCA) algorithm. Here, we default to setting $k=6$ for the most similar spots.

#### Shuffled graph

For the shuffled graph $\mathbf{G}_{\text{shuffled}}(\mathbf{X}_{\text{shuffled}}, \mathbf{E}_{\text{spa}})$, we adopt a perturbation operation that is similar to the technique used in GraphST and SpaceFlow. Here, $\mathbf{X}_{\text{shuffled}}$ represents the matrix of shuffled gene expression profiles.

### Constructing contrastive views

MuCoST constructs three contrastive views using the above three graphs, including the spatial view, positive view and repulsive view.

To elaborate further, the spatial view, denoted as $\mathbf{V}_{\text{spa}}(\mathbf{X}, \mathbf{E}_{\text{spa}})$, is derived from the spatial graph $\mathbf{G}_{\text{spa}}(\mathbf{X}, \mathbf{E}_{\text{spa}})$. To obtain the positive view $\mathbf{V}_{\text{pos}}(\mathbf{X}_{\text{mask}}, \mathbf{E}_{\text{spa}}||\mathbf{E}_{\text{coe}})$, we use two argumentation strategies. Concatenation of the edge list $\mathbf{E}_{\text{spa}}||\mathbf{E}_{\text{coe}}$ is performed along the last dimension where || represents the concatenation operator. $\mathbf{X}_{\text{mask}}$ denotes the random feature masking operation applied to gene expression profile $\mathbf{X}$. This masking technique can introduce variability into the positive view. Additionally, the repulsive view $\mathbf{V}_{\text{rep}}(\mathbf{X}_{\text{shuffled}}, \mathbf{E}_{\text{spa}})$ is directly derived from $\mathbf{G}_{\text{shuffled}}(\mathbf{X}_{\text{shuffled}}, \mathbf{E}_{\text{spa}})$. The repulsive view is used to prevent features from collapsing and to push away spots with significantly different features for a certain distance in the representation space.

### MPNN-based GCN autoencoder

MuCoST utilizes a shared graph convolutional autoencoder network to learn the latent representations. The encoder employs an MPNN-based GCN layer [37]. The MPNN framework is highly scalable and well suited for concatenating the operation of different graphs. The MPNN-based GCN encoder is formalized as 


(1) \begin{align*}& \mathbf{z}^{\prime}_{i}=\sum_{j \in \mathcal{N}(i)\bigcup{i}}\frac{w_{i,j}}{\sqrt{\tilde{d_{j}}\tilde{d_{i}}}}\mathbf{\Theta}_{e}\mathbf{x}_{j}+\mathbf{b}_{e},\end{align*}

where $\mathcal{N}(i)$ denotes the neighbor set, which can be deduced from edge list $\mathbf{E}$ of spot $\mathbf{x}_{i}$ that corresponds to a row of gene expression profiles $\mathbf{X}$, $\mathbf{z}^{\prime}_{i}$ represents the latent representation of spot $\mathbf{x}_{i}$, $\mathbf{x}_{j}$ denotes the neighbor gene expression of spot $\mathbf{x}_{i}$, $\mathbf{\Theta }_{e}$ represents the trainable weight matrix, $ \mathbf{b}_{e} $ represents the trainable bias, $w_{i,j}$ indicates the edge weight between spot $\mathbf{x}_{i}$ and $\mathbf{x}_{j}$ and $\tilde{{d}}_{i}$ and $\tilde{{d}}_{j}$ represent the degree of spot $\mathbf{x}_{i}$ and $\mathbf{x}_{j}$, respectively. Formally, $\tilde{{d}}_{i}=|\mathcal{N}(i)\bigcup{i}|$, in which $|\cdot | $ represents the number of elements within a set.

The decoder utilizes GCN layer to convert the latent representation back to the original feature space. The decoding component is formalized as 


(2) \begin{align*}& \mathbf{x}^{\prime}_{i}=\sum_{j \in \mathcal{N}(i)\bigcup{i}}\frac{w_{i,j}}{\sqrt{\tilde{d_{j}}\tilde{d_{i}}}}\mathbf{\Theta}_{d}\mathbf{z}^{\prime}_{j}+\mathbf{b}_{d},\end{align*}

where $\mathbf{x}^{\prime}_{i}$ is the reconstructed representation of spot $\mathbf{x}_{i}$.

We feed three views into the model, resulting in three corresponding latent representations: $\mathbf{Z}_{\text{spa}}$, $\mathbf{Z}_{\text{pos}}$ and $\mathbf{Z}_{\text{rep}}$. The encoder weights of parameters are shared across the three views, and the latent representation $\mathbf{Z}_{\text{spa}}$ is reconstructed via the decoder, yielding the reconstructed representation $\mathbf{X}^{\prime}$. Finally, to obtain a concise latent representation from the GCN autoencoder, the reconstruction loss is computed as 


(3) \begin{align*}& \mathcal{L}_{\text{Rec}}=\frac{1}{N}\sum_{i=1}^{N}\|\mathbf{x}^{\prime}_{\text{i}}-\mathbf{x}_{\text{i}}\|_{2}^{2},\end{align*}

where $N$ is the quantity of spots.

### Multi-view graph CL framework

To improve the model’s efficiency, the InfoNCE loss function [38] is utilized to learn discriminative information from the latent representation of three views. Before calculating loss, a sequence of batch normalization and nonlinear activation functions is applied to three latent representations as 


(4) \begin{align*}& \mathbf{Z}^{\prime}=\sigma(\text{BN}(\mathbf{Z})),\end{align*}

in which the ELU activation function $\sigma (\cdot )$ is employed on latent representations. Furthermore, to expedite convergence, batch normalization [39] $\text{BN}(\cdot )$ is utilized, and it can be formulated as 


(5) \begin{align*}& \mathbf{z}^{\prime}_{i}=\frac{\mathbf{z}_{i}-\text{E}[\mathbf{Z}]}{\sqrt{\text{Var}[\mathbf{Z}] + \epsilon}}\odot\mathbf{\gamma}+\mathbf{\beta},\end{align*}

where $\text{E}[\mathbf{Z}]$ denotes the mean of $\mathbf{Z}$, $\text{Var}[\mathbf{Z}]$ represents the variance, $\epsilon $ serves as a small number to avoid division by 0 and $\mathbf{\gamma }$ and $\mathbf{\beta }$ are the trainable weight matrix and bias vector, respectively.

The InfoNCE loss function is defined as 


(6) \begin{align*}& \mathcal{L}_{\text{InfoNCE}}=-\frac{1}{N}\sum_{i=1}^{N} \log\frac{\exp(\text{Sim}(\mathbf{z}^{\prime}_{\text{spa},i}, \mathbf{z}^{\prime}_{\text{pos},i})/\tau)}{\sum_{j=1}^{N}\exp(\text{Sim}(\mathbf{z}^{\prime}_{\text{spa},i},\mathbf{z}^{\prime}_{\text{rep},j})/\tau)},\end{align*}

where $\text{Sim}(\mathbf{z}^{\prime}_{\text{spa},i}, \mathbf{z}^{\prime}_{\text{pos},i})$ in the numerator represents the cosine similarity between $\mathbf{z}^{\prime}_{\text{spa},i}$ and $\mathbf{z}^{\prime}_{\text{pos},i}$, and so is the corresponding term $\text{Sim}(\mathbf{z}^{\prime}_{\text{spa},i}, \mathbf{z}^{\prime}_{\text{rep},j})$ in the denominator. The cosine similarity in the numerator can be formulated as 


(7) \begin{align*}& \text{Sim}(\mathbf{z}^{\prime}_{\text{spa},i}, \mathbf{z}^{\prime}_{\text{pos},i})=\frac{\mathbf{z}^{\prime}_{\text{spa},i}\cdot\mathbf{z}^{\prime}_{\text{pos},i}}{||\mathbf{z}^{\prime}_{\text{spa},i}||\cdot||\mathbf{z}^{\prime}_{\text{pos},i}||}.\end{align*}

By maximizing cosine similarity between $\mathbf{z}^{\prime}_{\text{spa},i}$ and $\mathbf{z}^{\prime}_{\text{pos},i}$, the model can extract consistent structural information from the two positive graphs. However, by minimizing the sum of cosine similarity between $\mathbf{z}^{\prime}_{\text{spa},i}$ and all spots in $\mathbf{Z}^{\prime}_{\text{rep}}$, $\mathbf{Z}^{\prime}_{\text{spa}}$ will avoid collapsing into representations where all spots are perceived as similar. $\tau $ is the temperature coefficient. We empirically set $\tau = 0.05$ for 10X Visium data, and we set $\tau = 1$ in Stereo-seq data with cellular resolution.

To optimize the model, we minimize a total loss function as 


(8) \begin{align*}& \min\mathcal{L}_{\text{Total}}=\min(\mathcal{L}_{\text{Rec}}+\lambda\cdot\mathcal{L}_{\text{InfoNCE}}),\end{align*}

where the weight parameter $\lambda $ serves as a trade-off factor of the model’s ability to adjust CL. We empirically set $\lambda =0.2$, which yields the desired performance.

### Clustering and downstream analysis

We perform subsequent analysis on the latent representation $\mathbf{Z}_{\text{spa}}$ following training. Initially, we use the PCA algorithm to identify the principal components and keep the dimension of latent representations. Then, we apply the mclust [40] algorithm from R and Leiden and Louvain algorithm from Scanpy to generate category labels for all spots. Additionally, for human DLPFC of the 10X Visium benchmark datasets, we leverage the refine operation to reassign category labels, which are consistent with the majority of default $r=25$ neighbors in the spatial map (see Figure S5 for refinement results). Upon clustering, we make inferences of spatial trajectory using PAGA in Scanpy. We decipher the spatial architectures of a tissue slice using the spatial display tools in Scanpy. We use Ripley’s L function in Squidpy to calculate the spatial aggregation or discrepancy pattern of the spatial domains [32]. We use the gene ranked tool in Scanpy to identify spatially variable genes. In addition, we visualize the up-regulated signaling pathways in the functional spatial domains through GO enrichment analysis by GSEApy [35].

**Key Points**

- We present MuCoST, a multi-view graph CL framework for deciphering complex spatially resolved transcriptomic architectures.
- The key innovation of MuCoST is that it allows to learn relatively consistent representation from dual scale structural dependency, and encourages the model to learn discriminative representation from random expression, thus making the model learn more complete structural information.
- MuCoST detects spatial domains through clustering in the latent representation, and it can perform analytical tasks that include spatial cluster visualization, spatial domain identification, PAGA trajectory inference, subtle biological texture recognizing and functional analysis of spatial domain from spatial gene expression heterogeneity.
- The results show that MuCoST is superior to the competing methods in terms of spatial domain identification accuracy and clustering representation of compactness and separability.
- In particular, MuCoST accurately captures subtle biological textures from fine-grained spatial domains. By incorporating differential gene expression analysis and gene enrichment analysis, the learned spatial domains possess interpretability and exhibit specific biological functions.

## Supplementary Material

Supplementary_Information_bbae255

## Data Availability

**Code:** MuCoST software is available online at https://github.com/tju-zl/MuCoST**Data:** All datasets are open-access. The DLPFC dataset is available online at http://research.libd.org/spatialLIBD/. The coronal mouse brain of 10X Visium dataset is available online at https://squidpy.readthedocs.io/en/latest/notebooks/tutorials/tutorial_visium_hne.html. Mouse olfactory bulb of Stereo-seq dataset is available online at https://github.com/JinmiaoChenLab/SEDR_analyses. Human breast cancer of 10X Visium dataset is available online at https://support.10xgenomics.com/spatial-gene-expression/datasets/1.1.0.

## Author Biographies

**Lei Zhang** is currently pursuing the Ph.D. degree in control science and engineering from Tongji University, Shanghai, China. His research interests include computational biology and machine learning.

**Shu Liang** is a research professor in Tongji University, Shanghai, China. His research interests include intelligent information processing, machine learning and optimization theory.

**Lin Wan** is a professor in the Academy of Mathematics and Systems Science, Chinese Academy of Sciences. He is also a faculty member in the School of Mathematical Sciences at University of Chinese Academy of Sciences, China. His research focuses on computational biology, systems biology and data science.
