# Supplementary material for: A multi-view graph contrastive learning framework for deciphering spatially resolved transcriptomics data
Source: Brief Bioinform. 2024 May 27;25(4):bbae255. doi: 10.1093/bib/bbae255 (PMC11129769; doi:10.1093/bib/bbae255)
Supplement: Supplementary_Information_bbae255 [file supplementary_information_bbae255.pdf]

Supplementary Information

A Multi-view Graph Contrastive Learning Framework for Deciphering Spatially Resolved Transcriptomics Data

Lei Zhang[12], Shu Liang[12*], Lin wan[34*]

[1]Department of Control Science and Engineering, Tongji University, No. 4800 Cao'an Road, 201804, Shang Hai, China.

[2]Shanghai Research Institute for Intelligent Autonomous Systems, Tongji University, Lane 55, Chuanhe Road, 201210, Shang Hai, China.

[3]Academy of Mathematics and Systems Science, Chinese Academy of Sciences, No. 55 Zhongguancun East Road, 100190, Beijing, China.

[4]School of Mathematical Sciences, University of Chinese Academy of Sciences, 19A Yuquan Road, 100049, Beijing, China.

[*]Corresponding author. Lin Wan. Tel.: +86 1082541203; E-mail: lwan@amss.ac.cn.

[*]Corresponding author. Shu Liang. Tel.: +86 18604421682; Email: sliang@tongji.edu.cn.

This file includes the following subsections:

- **Evaluation Criteria**
- **Competing Methods**
- **Supplemental Figures**
- **Supplemental Note**

Evaluation Criteria

Adjusted Rand Index (ARI)

When benchmarking on DLPFC datasets, we calculate the ARI to evaluate the similarity between clustering labels and the real annotated labels, and the ARI is calculated as

$$\mathrm{ARI}=\frac{\mathrm{RI}-\mathbb{E}[\mathrm{RI}]}{\max(\mathrm{RI})-\mathbb{E}[\mathrm{RI}]},$$

where the Rand index RI compares whether each spot in the clustering result and annotation belongs in the same category, and $\mathbb{E}[\mathrm{RI}]$ is the expected value of RI. ARI ranges from 0 to 1 where 0 represents random similarity between clustering label and real annotated label, and 1 means equality. By comparing the ARI of different methods on benchmark datasets, performance can be evaluated.

Normalized mutual information (NMI)

NMI measures the similarity between two clusters. NMI is an extension of mutual information (MI) which measures the amount of information shared between two random variables. NMI normalizes the MI score to a range between 0 and 1, making it easier to interpret and compare across different datasets. NMI is calculated as

$$\mathrm{NMI}=\frac{2\times \mathrm{MI}(\mathrm{Y},\mathrm{Y}')}{\mathrm{H}(\mathrm{Y})\mathrm{H}(\mathrm{Y}')},$$

where Y and Y' represent ground truth and clustering distribution, $\mathrm{MI}(\mathrm{Y},\mathrm{Y}')$ denotes mutual information between Y and Y', and $\mathrm{H}(\mathrm{Y})$ and $\mathrm{H}(\mathrm{Y}')$ represent the entropies of Y and Y', respectively.

Homogeneity score and completeness score (HS)

Homogeneity score (HS) quantifies the degree to which clusters contain only spots that belong to a single category. Homogeneity is based on the concept of clusters purity. A cluster is considered homogeneous if it contains data points from only one true class. HS measures the extent to which this property holds across all clusters in the clustering result. HS is calculated as

$$\mathrm{HS}=1-\frac{\mathrm{H}(\mathrm{Y}|\mathrm{Y}')}{\mathrm{H}(\mathrm{Y})},$$

where $\mathrm{H}(\mathrm{Y}|\mathrm{Y}')$ is the conditional entropy of the true class labels given the cluster assignments, and $\mathrm{H}(\mathrm{Y})$ is the entropy of the true class labels. HS ranges from 0 to 1 where 1 indicates perfect homogeneity, meaning that each cluster contains only data points from a single class. A score close to 0 indicates low homogeneity, indicating that the clusters are mixed or poorly separated with respect to true class labels.

Completeness score (CS) measures the extent to which all samples of a given class are assigned to the same cluster. It quantifies the degree to which clustering covers all samples from a particular class. CS ranges from 0 to 1, and a value of 1.0 indicates perfect completeness, which means that all samples of a class are assigned to the same cluster. Lower values indicate that samples from a class are split across multiple clusters, indicating incomplete coverage. CS is calculated as

$$\mathrm{CS}=1-\frac{\mathrm{H}(\mathrm{Y}'|\mathrm{Y})}{\mathrm{H}(\mathrm{Y}')}.$$

V_measure

V_measure is a metric used to evaluate the quality of clustering algorithms. It combines

homogeneity and completeness measures into a single score. It measures the amount of uncertainty in the clustering results and the extent to which clustering accurately represents true classes or labels. $\mathrm{V\_measure}$ ranges from 0 to 1 where a value of 1 indicates a perfect clustering result with high homogeneity and completeness. $\mathrm{V\_measure}$ score is calculated as

$$\mathrm{V}_{\mathrm{measure}} = (1+\beta) \cdot \frac{\mathrm{HS} \cdot \mathrm{CS}}{\beta \cdot \mathrm{HS} + \mathrm{CS}},$$

where the weight of β is attributed to homogeneity with completeness. If $\beta = 1$, $\mathrm{V\_measure}$ is equal to NMI. If β is greater than 1, completeness is weighted more strongly in the calculation. If β is less than 1, homogeneity is weighted more strongly. We evaluate β with values of 0.5 and 1.5.

Silhouette coefficient score (SC)

SC is a clustering analysis criteria without ground truth. SC considers not only the compactness within clusters, but also the separation between clusters. The value range of SC is between $[-1, 1]$, and the larger the score, the better the clustering result. SC score for a spot is calculated as

$$\mathrm{SC} = \frac{b-a}{\max(a,b)},$$

where a is the mean intra cluster distance and b is the mean nearest-cluster distance for each spot.

Davies-Bouldin Index (DB)

DB is another clustering analysis criteria without ground truth. It measures the average similarity between clusters, taking into account both the intra-cluster similarity and the inter-cluster dissimilarity. DB score is calculated as

$$\mathrm{DB} = \frac{1}{K} \sum_{i,j=1}^{K} \max_{i \neq j} \frac{s_i + s_j}{d_{ij}},$$

where K is the number of clusters, s_i is the average dissimilarity between each spot in the cluster and the centroid of the cluster and d_{ij} is the distance between the centroid of the cluster i and the centroids of cluster j.

All the above evaluation criteria scores are calculated by Scikit-learn.

Competitive Methods

Criterion scores of all methods are evaluated through batch testing on DLPFC datasets, with a fixed seed of 2023. Furthermore, to ensure a fair evaluation of the representation learning performance of the methods, we remove the classification label refinement function from all methods. For other datasets, we follow the tutorials provided by each method.

Scanpy

We followed Scanpy's tutorials and compared the performance on DLPFC datasets (https://scanpy.readthedocs.io/en/stable/tutorials.html). To be specific, we first adopted preprocessing methods consistent with MuCoST, then used PCA to reduce the dimension of gene expression profile, and finally used Leiden algorithm for clustering. In particular, we did not use the spatial information for representation learning.

SpaGCN

SpaGCN uses extra histological information to enhance spatial information, and we followed its tutorial to evaluate on DLPFC datasets, including mouse brain posterior tissue and coronal mouse brain section (https://github.com/jianhuupenn/SpaGCN/blob/master/tutorial/tutorial.ipynb). In addition, in order to compare the performance of representation learning fairly, we canceled the label refine function of SpaGCN in DLPFC datasets.

STAGATE

We used the STAGATE version based on PyG framework (https://pytorch-geometric.readthedocs.io/en/latest/index.html), which is also adopted in MuCoST. We followed STAGATE's tutorials and recommended hyperparameters to evaluate all datasets (Tutorials of STAGATE: https://stagate.readthedocs.io/en/latest/index.html).

GraphST

GraphST is a contrastive learning framework based on deep graph infomax (DGI), and we followed its tutorials to evaluate all datasets (https://deepst-tutorials.readthedocs.io/en/latest/index.html). In addition, in order to compare the performance of representation learning fairly, we canceled the label refinement function of GraphST in DLPFC datasets.

SpaceFlow

SpaceFlow uses the DGI framework to learn the latent representation of SRT data, and its purpose is to reveal the spatiotemporal patterns of cells based on pseudo-spatiotemporal map. We followed its tutorial to evaluate the spatial clustering performance on the DLPFC dataset (https://github.com/hongleir/SpaceFlow/blob/master/tutorials/seqfish_mouse_embryogenesis.ipynb).

ConST

ConST is an interpretable multi-modal method based on the DGI framework. We followed its tutorial to evaluate DLPFC datasets, including mouse brain posterior tissue and coronal mouse brain section (https://github.com/ys-zong/conST/blob/main/conST_cluster.ipynb).

Supplemental Figures

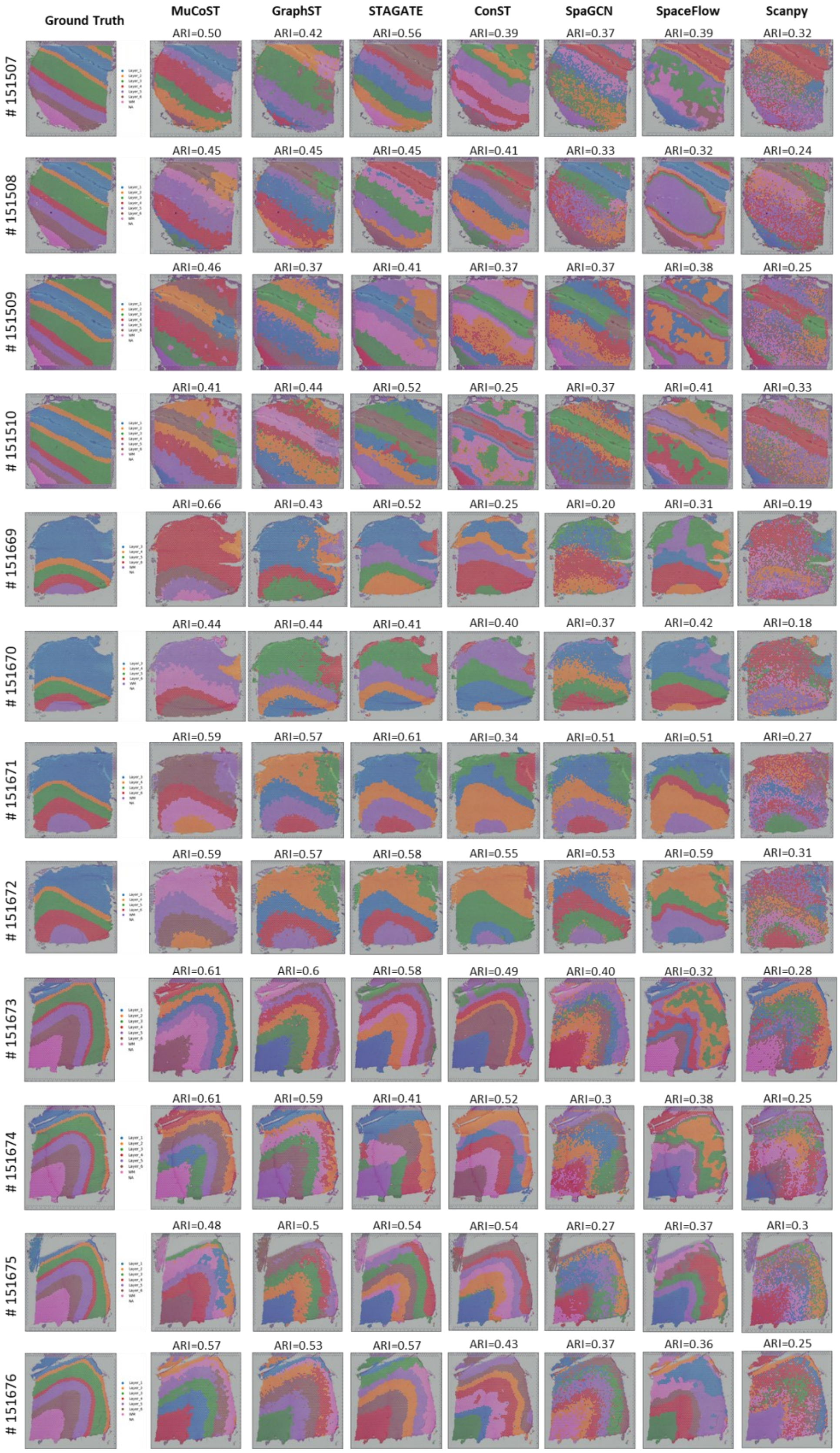

Fig.S1. Comparison of spatial domains identification on 12 slices of DLPFC datasets by MuCoST, GraphST, STAGATE, ConST, SpaGCN, SpaceFlow and Scanpy.

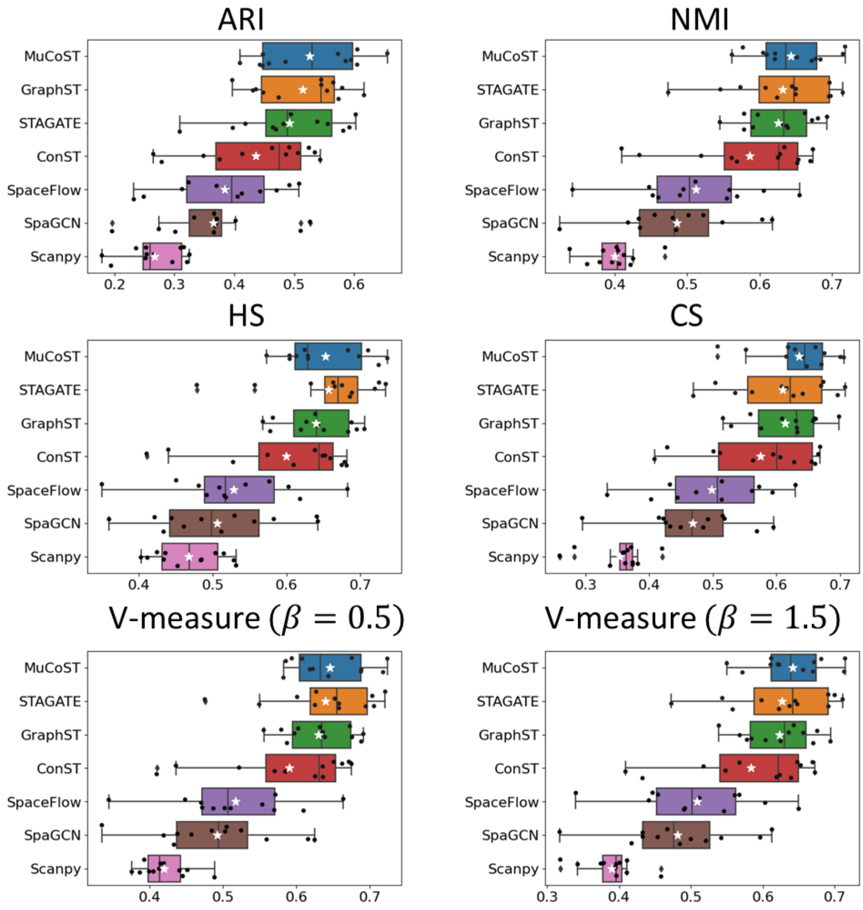

Fig.S2. Comparison of evaluation criteria with adjusted Rand index (ARI), normalized mutual information (NMI), homogeneity score (HS), completeness score (CS) and V-measure on 12 slices of DLPFC datasets by MuCoST, GraphST, STAGATE, ConST, SpaceFLow, SpaGCN and Scanpy.

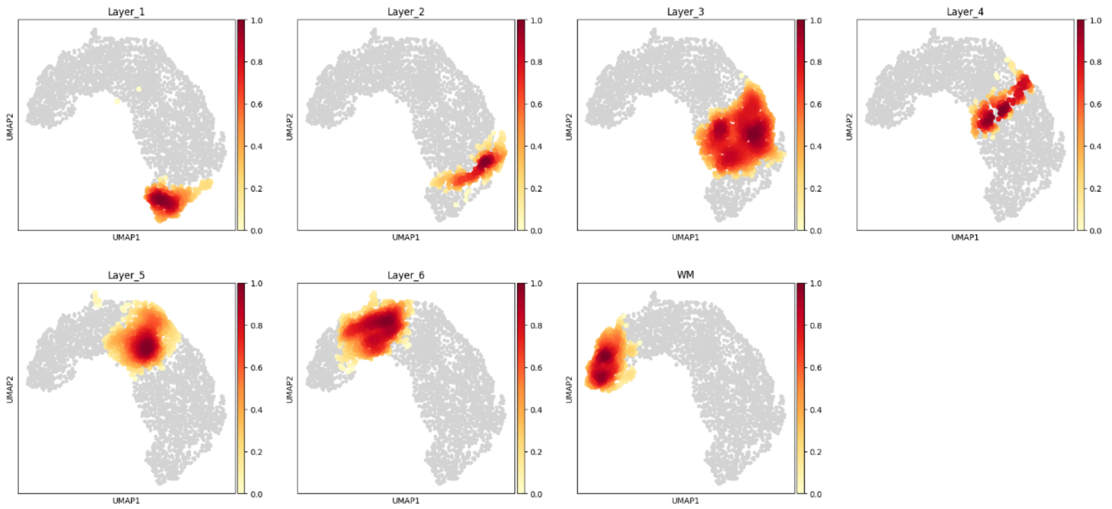

Fig.S3. Density heatmap of each cluster on DLPFC dataset slice #151673 based on UMAP.

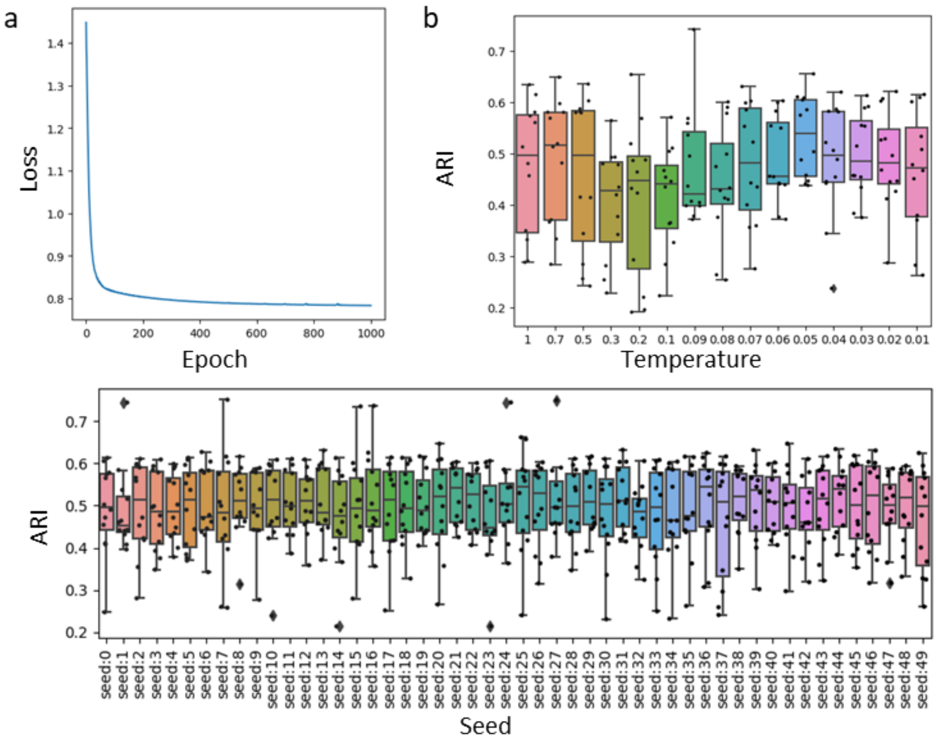

Fig.S4. Robust analysis of MuCoST. a. Loss curve of MuCoST training on DLPFC dataset slice #151673. **b.** ARI of MuCoST on all 12 sections of DLPFC datasets with different temperature in InfoNCE loss function. **c.** ARI of MuCoST in all 12 sections of DLPFC datasets with different seed.

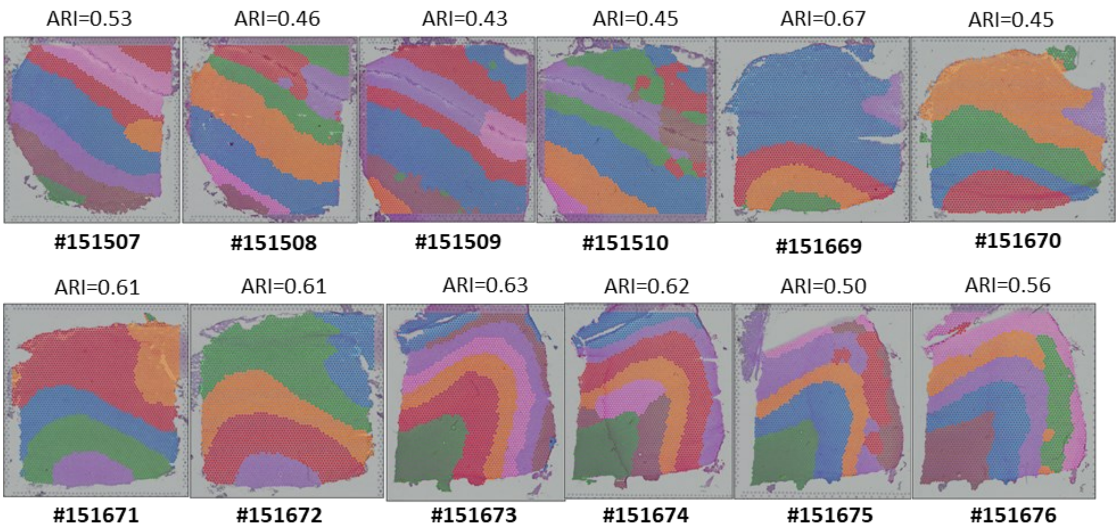

Fig.S5. MuCoST using spatial refine to improve the accuracy of spatial clustering.

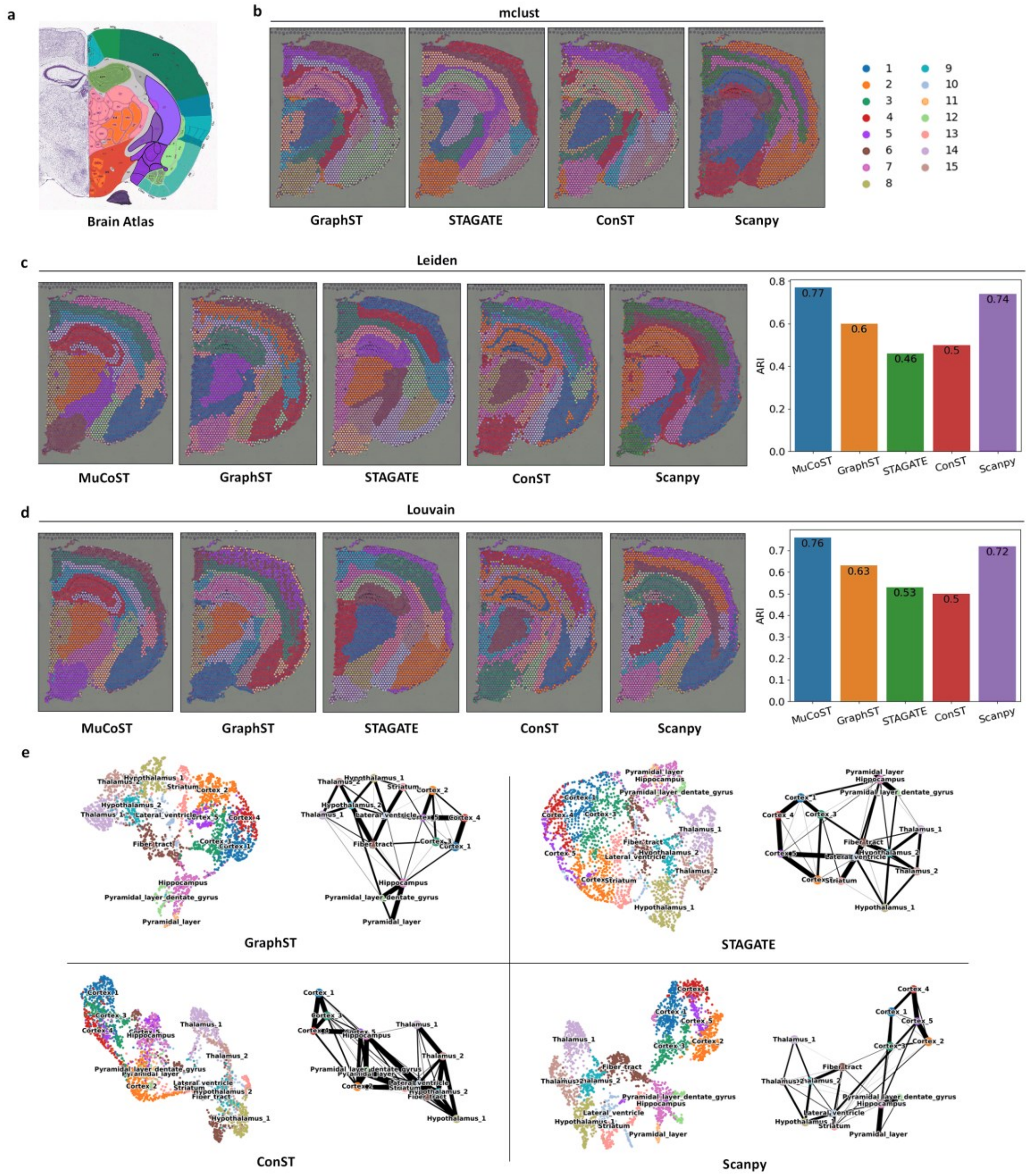

Fig.S6. Comparison of subtle biological textures recognition of mouse brain tissues by different methods. a. Atlas of Allen mouse brain. **b.** Results of competitive methods based on mclust clustering algorithm. **c.** Results of different methods based on Leiden clustering algorithm with domain=15. **d.** Results of different methods based on Louvain clustering algorithm with domain=15. **e.** Results of different methods on spatial visualization and PAGA trajectory inference.

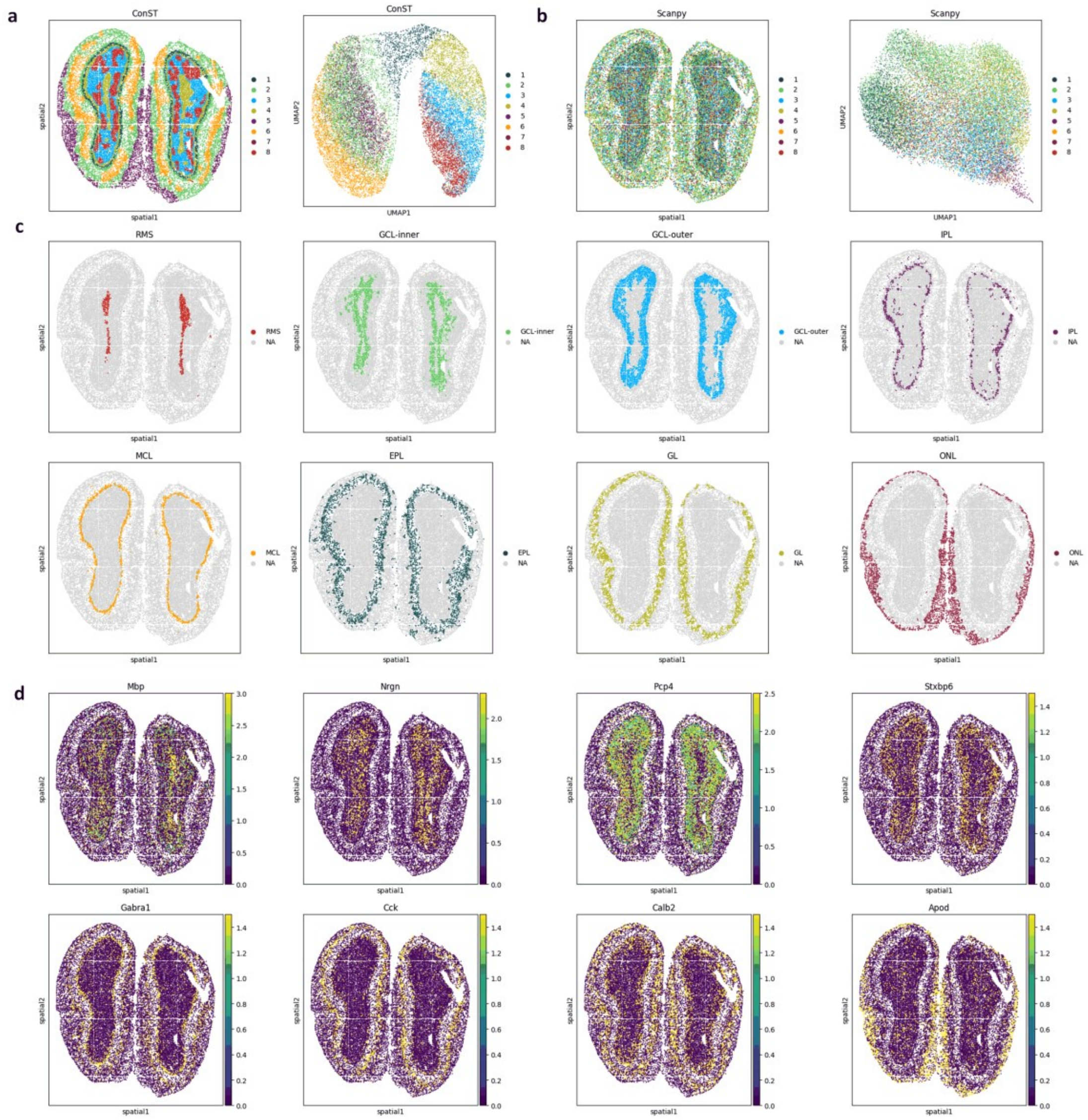

Fig.S7. MuCoST accurately identifies the laminar organization of mouse olfactory bulb. a. The spatial domain identified by ConST and UMAP visualization. **b.** The spatial domain identified by Scanpy and UMAP visualization. **c.** Visualization of corresponding annotated layers based on spatial domain identification of MuCoST. **d.** Expression of marker genes in all laminar organizations.

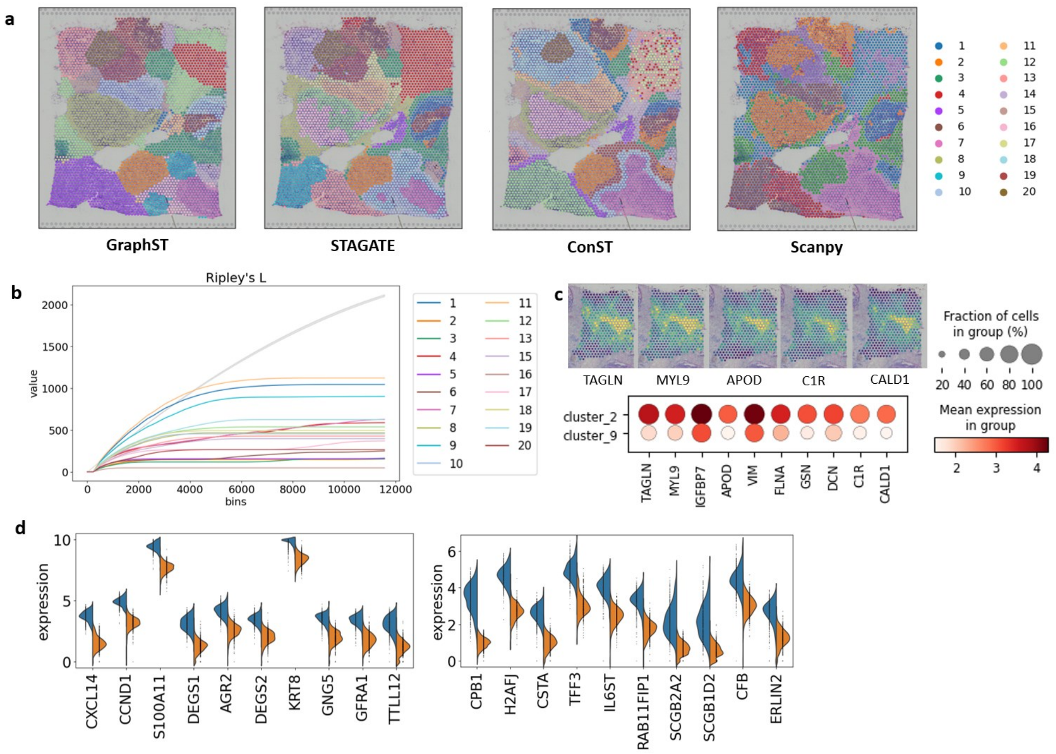

Fig.S8. MuCoST captures spatial heterogeneity more accurately. a. Comparison of spatial domain identification results of competitive methods. **b.** Calculating the clustered or dispersed of spots distribution based on Ripley's L function. **c.** Spatial variable genes visualization in spatial domain 2 and point plot of gene differential expression between spatial domain 2 and spatial domain 9. **d.** Differential expression of spatially variable genes in spatial domain 1 and spatial domain 11.

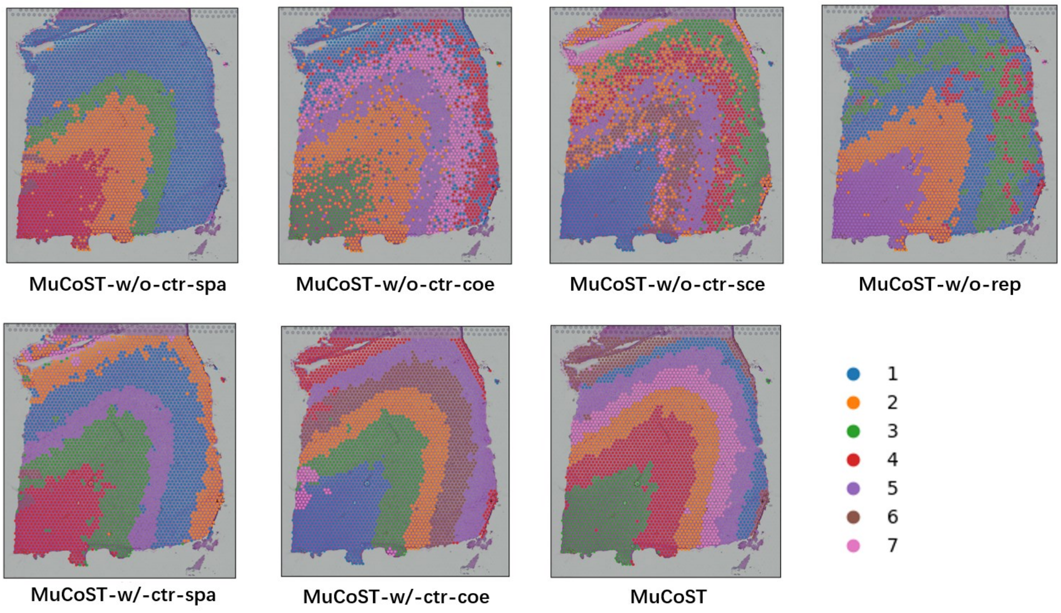

Fig.S9. Comparison of spatial domain identification results of MuCoST and its variants on the slice #151673 of DLPFC dataset.

Supplemental Note

Hyperparameter of MuCoST

MuCoST uses the following default hyperparameters (Tab.S1).

Table.S1. Default hyperparameters of MuCoST

Setting	Default value
Seed	2023
Epoch	1000
Learning rate	0.001
Weight decay	0.
GCN layer	Encoder=1; Decoder=1
Latent dim	50
Radius KNN (Set by spatial resolution)	150/300 (10X); 50 (Stereo-seq)
Maximum neighbor of KNN	6
Refine labels	K=25
Temperature (InfoNCE)	0.05 (10X); 1 (Stereo-seq)
Probability of feature dropout	0.2
Flow of message passing	Source to target
